# Differences in mental health status between individuals living with diabetes, and pre-diabetes in Qatar: A cross-sectional study

**DOI:** 10.1016/j.heliyon.2023.e23515

**Published:** 2023-12-09

**Authors:** Montaha Mahmoud, Razi Mahmood

**Affiliations:** a College of Health Sciences, University of Doha for Science and Technology, Doha, Qatar; b AFG College with the University of Aberdeen, Doha, Qatar

**Keywords:** Diabetes, Prediabetes, Depression, Anxiety, Qatar, Epidemiology

## Abstract

**Aims:**

The aims of this study was to determine the prevalence and to compare depression and anxiety screening scores by type of diabetes: type 2 diabetes mellitus (T2DM), type 1 diabetes mellitus (T1DM), and pre-diabetes. The secondary aim was to examine sex differences in screening scores by type of diabetes.

**Methods:**

This cross-sectional study was conducted in Doha, Qatar using primary data collection (N = 150), and stratified random sampling at a diabetes primary healthcare center. The study tool collected demographic information and used validated mental health screening tools for depressive symptoms “Patient Health Questionnaire-9 (PHQ-9)", and for anxiety symptoms “Generalized Anxiety Disorder 7 (GAD-7)".

**Results:**

The prevalence of moderate to severe depressive and anxiety scores was highest in the prediabetes group (20 % and 14 % respectively). There were increased PHQ-9 scores in the pre-diabetes group compared to T2DM (p-value <0.05). No statistically significant differences in depressive symptom scores were found when comparing the pre-diabetes group with T1DM, and T1DM with T2DM. When looking at sex differences, there were no statistically significant differences between T1DM and pre-diabetes males and females, however PHQ-9 and GAD-7 scores in T2DM females were poorer compared to T2DM males.

**Conclusion:**

The results of our study found patients living with pre-diabetes, and females with T2DM are vulnerable populations who should be screened for mental health disorders. Early screening for mental health disorders for individuals diagnosed with prediabetes, T1DM, and T2DM should be routinely conducted to potentially improve health outcomes.

## Abbreviations

T1DM: Type 1 Diabetes Mellitus
T2DM: Type 2 Diabetes Mellitus
PHQ-9: Patient Health Questionnaire-9
GAD-7: Generalized Anxiety Disorder-7

## 1 Introduction

The World Health Organization (WHO) defines diabetes as a “chronic metabolic disease characterized by elevated levels of blood glucose” [1]. There are 2 main types of diabetes, type 1 diabetes mellitus (T1DM), and type 2 diabetes mellitus (T2DM) [2]. T1DM is when the body does not produce insulin as a result of an autoimmune reaction due to an unknown cause [2]. T2DM is diagnosed when the body does not use the insulin produced effectively to control blood sugar levels [2]. T2DM is more prevalent than T1DM and accounts for approximately 90–95 % of diabetes cases [2]. Pre-diabetes is one of the main risk factors associated with the development of T2DM [3]. It is defined as a state of impaired glucose tolerance and impaired fasting glucose where blood glucose levels are elevated above the normal range but below the threshold to be clinically diagnosed with T2DM [3][4].

According to the International Diabetes Federation (IDF), in 2021, the Middle East and North Africa (MENA) region had the highest age-adjusted comparative prevalence of diabetes (18.1 %) among individuals aged between 20 and 79 years old, compared to 11.2 % globally [5]. In addition, the MENA region reported the highest percentage of diabetes-associated mortality (24.5 %) among adults aged up to 60 years old [5]. Moreover, projections indicate that by 2045 the estimated comparative prevalence of diabetes in the MENA region will increase to 20.4 % indicating diabetes is a public health concern that continues to grow [5].

The increased blood glucose levels seen among individuals with diabetes can cause serious damage to the body if not managed appropriately [1]. Almost every part of the human body may be negatively affected including the heart, kidney, nervous system, oral cavity, blood vessels, and mental health [[6], [7]]. Mental health disorders, such as depression and anxiety can affect the way a person functions in their daily activities [8]. The Canadian Clinical Diabetes Guideline (2018) stated that people living with diabetes may be at a higher risk of depression, particularly individuals who are female, diagnosed with diabetes during adolescence, low socioeconomic status, little social support, longer duration of diabetes, poor glycemic control, higher illness burden, stressful life events and the presence of long-term complications [9]. This finding is troubling as depression can negatively affect the way individuals manage diabetes, increasing the risk of developing more severe diabetes complications [10]. Similar to depression, anxiety has been identified as a common co-morbid condition with generalized anxiety disorder (GAD) being the most common form of anxiety disorders seen among T2DM patients [11]. Disconcertingly, anxiety has been found to be an independent risk factor for individuals with pre-diabetes to progress to T2DM patients [12,13]. A recent longitudinal study concluded that at 3 months and 12 months post-follow up, T2DM patients with anxiety disorders had poorer glycemic control compared to T2DM patients without any anxiety disorder [14]. These findings on the relationship between depression and anxiety with diabetes are consistent with the Canadian Diabetes Association (CDA) Clinical Practice Guidelines which reported that people living with diabetes and mental health disorders have lower medication adherence than those without mental health disorders [15]. Consequently, decreased diabetes self-care compliance leads to a higher risk of functional impairment and an increased risk of complications associated with diabetes resulting in decreased quality of life, increased risk of early mortality and increased healthcare costs for managing diabetes [15].

In Qatar, it is estimated that approximately 20 % of patients presenting to Primary Health Care Centers (PHCC) reported at least one mental health disorders with major depression disorders, and anxiety disorders being the most common [16]. Among T2DM patients, approximately 20 % of T2DM patients attending family medicine clinics in Qatar were diagnosed with depression [17]. In addition, another study indicated that T2DM patients had higher proportions of severe depression and severe anxiety scores compared to healthy controls in Qatar [18]. There is however, a dearth of information examining the prevalence of depression or anxiety among T1DM patients or individuals with pre-diabetes in Qatar, nor when comparing between groups.

The purpose of this cross-sectional study was to determine the prevalence of depression and anxiety (outcome) using screening tools based on the type of diabetes (T2DM, T1DM, prediabetes) (exposure), and to investigate the relationship between the type of diabetes (exposure) and mental health outcomes (anxiety and depression) among individuals living in Qatar. The secondary objective of this study was to examine sex differences in mental health screening scores by type of diabetes.

## 2 Methods

### 2.1 Study design

This study used a cross-sectional study design with primary data collection. The study design was appropriate as it enabled us to determine the prevalence of depression and anxiety (outcomes) based on exposure status (type of diabetes/pre-diabetes) in a relatively quick, and cost-effective manner [19] for which there is a lack of information in Qatar. Moreover, we were also able to further investigate outcomes based on sex differences. Ethics approval to conduct this research using anonymized surveys collected by telephone interviews was approved by the Georgetown in Qatar Institutional Review Board (IRB) (Study00004668), and all participants provided oral informed consent.

### 2.2 Study tool

The study tool was a survey consisting of demographic information, the Patient Health Questionnaire-9 (PHQ-9), and the Generalized Anxiety Disorder-7 (GAD-7). The validated questionnaires (PHQ-9, and GAD-7) have been used in the Middle East and North Africa in previous studies, indicating their validity as appropriate tools for this study [20,21].

### 2.3 Setting

Data collection was conducted at the Qatar Diabetes Association (QDA) in Doha, Qatar. The QDA was established in 1995 to support adults, children, and families living with diabetes to achieve diabetes control and avoid complications [22]. Participant recruitment and data collection were conducted at QDA between April 2022 to June 2022. Patients living with type 2 diabetes, and type 1 diabetes, and individuals in the pre-diabetes stage provided informed consented orally prior to their enrollment in this study.

### 2.4 Participants

Participants were identified using the QDA's patient records with a diagnosis of type 1 diabetes, type 2 diabetes, or pre-diabetes. Individuals eligible for this study included those aged 18–50 years old and had less than three co-morbidities (to limit confounder effects on depression or anxiety). Individuals who were previously diagnosed or treated for a mental health disorder were excluded from this study and those with three or more co-morbidities.

G*Power software version 3.1.9.6. was used to calculate the sample size needed to power the study assuming 80 % power, a 5 % level of significance, and a low effect size (f = 0.25). As a result, the sample size was calculated to be n = 159, or approximately 53 per study group. Stratified random sampling was used to select the participants (to ensure sufficient individuals with type 1 diabetes and pre-diabetes, as the majority of participants had type 2 diabetes). A list of all potential candidates was provided by the QDA. Initial stratification was based on the three exposure groups: type 1 diabetes, type 2 diabetes, and pre-diabetes. The Epitools epidemiological calculator was used to randomly select 50 individuals from each of the 3 exposure groups [23].

**Fig. 1:**
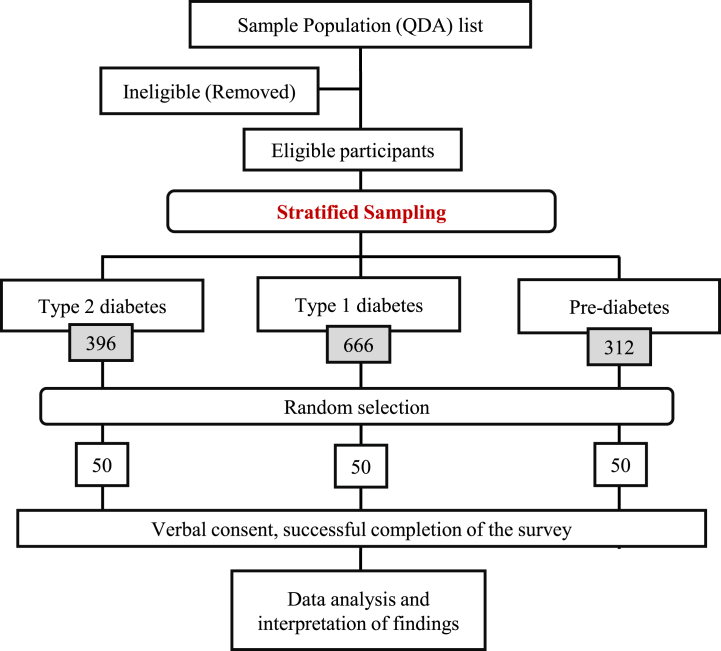
The process of enrollment for the study participants.

The individuals selected were then contacted to participate in the study. Data collection was conducted with the interviewer contacting the participants over the phone and after receiving consent or re-scheduling the phone call recording their responses on surveys without any identifiers of the participant. Interviews were conducted in English or Arabic by the same interviewer depending on the participant's preference. Prospective participants were contacted three times, in the morning, afternoon, and evening on nonconsecutive days for the duration of the data collection period. In case of non-response, additional participants were selected using the same approach however the individuals previously selected were removed from the stratified random sampling process (Fig. 1).

### 2.5 Variables

The study variables for this project include baseline demographic information (sex, age, nationality, highest level of educational attainment, marital status, body mass index (BMI), duration of diabetes (years), availability of family support, employment status, smoking status, number of comorbidities, family history of diabetes mellitus), the exposure variable which is type of diabetes (pre-diabetes, T1DM, T2DM) and the outcome variables, the 9-items Patient Health Questionnaire-9 (PHQ-9) scores and the 7-items General Anxiety Disorder (GAD-7) scores.

#### 2.5.1 Exposure variables

The exposure variable (type of diabetes) was previously diagnosed in a healthcare facility (primary health care), after which they were referred to the QDA for consultation. Individuals were classified based on national standards for the diagnosis of diabetes which is the same as the American Diabetes Association (ADA) standards as follows: Pre-diabetes: Glycated hemoglobin (HbA1C) of 5.7 %–6.4 %, Fasting blood glucose (FPG) between 100 and 125 mg/dL or Oral Glucose Tolerance Test (OGTT) 2 h blood glucose of 140–199 mg/dL; T1DM or T2DM: HbA1C of 6.5 % or greater, FPG of 126 mg/dL or greater, 2-h PG of 200 mg/dL or greater and Type 2 Diabetes (HbA1C = 6.5 % or higher) [24]. Additional screening tests for autoimmune markers such as islet cell autoantibodies, insulin autoantibodies as well as glutamic acid decarboxylase antibodies or c-peptide test as per the case and guidelines if required [24].

#### 2.5.2 Outcome variables

The outcome variables (PHQ-9 and GAD-7 scores) were calculated and categorized as follow. The PHQ-9 scores were calculated by assigning scores of 0, 1, 2, and 3, to the response categories of “not at all”, “several days”, “more than half the days”, and “nearly every day” to yield a final score of 0–27 (9 items). The PHQ scores were re-categorized to align with previous literature as follows: 0–4 indicating minimal depression severity, 5–9 indicating mild depression severity, and 10 or more indicating moderate to severe depression [25].

The GAD-7 anxiety severity scores were calculated by assigning scores of 0, 1, 2, and 3, to the response categories of “not at all”, “several days”, “more than half the days”, and “nearly every day” for a final score of 0–21 points (7 items). GAD-7 scores were re-categorized as follow: 0–4 none to minimal, 5–9 mild, 10–14 moderate, and 15–21 severe [26].

### 2.6 Statistical methods

Descriptive statistics for the study sample were summarized as frequencies and proportions for categorical variables and the mean and standard deviation for continuous variables stratified by exposure groups (T1DM, T2DM, and pre-diabetes).

Differences between exposure groups were calculated using the chi-squared test (χ2 test) and Fisher exact test for categorical variables and f-statistic (one-way ANOVA) for continuous variables. One-way ANOVA was used to assess whether differences existed in the mean PHQ-9 and GAD-7 scores between the exposure groups (T1DM, T2DM, and pre-diabetes). If a statistically significant difference was found, Tukey HSD post-hoc test was used to determine which exposure groups differed.

When analyzing sex differences between the groups, data was stratified by sex and differences in mean PHQ-9 and GAD-7 scores were examined using an independent samples *t*-test for each diabetes group as follows: T1DM male vs. female; T2DM male vs. female; and Pre-diabetes male vs. female. All data analysis was performed using (IBM SPSS Statistics for Mac, version 28 (IBM Corp., Armonk, N.Y., USA) with a p-value ≤0.05 indicating statistically significant results.

## 3 Results

### 3.1 Descriptive statistics

A total of 150 individuals were recruited for this study (50 with T1DM, 50 with T2DM, and 50 with pre-diabetes). Table 1 summarizes frequencies for categorical variables and the mean ± standard deviation for continuous variables across study groups (T1D, T2D, and pre-diabetes).

**Table 1:** Sample characteristics stratified by type of diabetes (N = 150).

Variable	Levels	Type 1 diabetes (n = 50)	Type 2 diabetes (n = 50)	Pre-diabetes (n = 50)	Total (N = 150)	p-value
**Sex**	Male Female	29 (58 %) 21 (42 %)	39 (78 %) 11 (22 %)	23 (46 %) 27 (54 %)	91 (60.7 %) 59 (39.3 %)	0.04
**Age**	Years	32.98 ± 7.59	42.10 ± 5.09	39.90 ± 5.88	150 (100 %)	<0.001
**Nationality**	Qatari Non-Qatari	9 (18 %) 41 (82.0 %)	0 (0 %) 50 (100.0 %)	2 (4.0 %) 48 (96.0 %)	11 (7.3 %) 139 (92.7 %)	0.001
**Education**	Less than Secondary	0 (0 %)	4 (8.2 %)	0 (0 %)	4 (2.7 %)	
	Secondary degree	13 (26 %)	14 (28.6 %)	8 (16 %)	35 (23.5 %)	
	Post-secondary education	2 (4 %)	11 (22.4 %)	3 (6 %)	16 (10.7 %)	<0.001
	University degree or higher	35 (70 %)	20 (40.8 %)	39 (78 %)	94 (63.1 %)	
**Marital status**	Single Divorced Engaged Married	12 (24 %) 3 (6 %) 0 (0 %) 35 (70 %)	2 (4 %) 0 (0 %) 1 (2 %) 47 (94 %)	4 (8 %) 1 (2 %) 0 (0 %) 45 (90 %)	18 (12 %) 4 (2.7 %) 1 (0.7 %) 127 (84.7 %)	0.004
**BMI**	Obesity Overweight Healthy weight	10 (20 %) 23 (46 %) 17 (34 %)	18 (39.1 %) 22 (47.8 %) 6 (13 %)	28 (56 %) 14 (28 %) 8 (16 %)	56 (38.4) 59 (40.4 %) 31 (21.2 %)	0.02
**Duration of diabetes**	Years	15.50 ± 8.13	6.43 ± 5.27	4.27 ± 3.02	150 (100 %)	<0.001
**Family support**	No Yes	24 (48 %) 26 (52 %)	30 (60 %) 20 (40 %)	39 (79 %) 10 (20.4 %)	93 (62.4 %) 56 (37.6 %)	0.005
**Employment status**	Employed	37 (74 %)	41 (82 %)	36 (72 %)	114 (76 %)	NS
	Unemployed	13 (26 %)	9 (18 %)	14 (28 %)	36 (24 %)	
**Smoking status**	Smoker	6 (12 %)	10 (20 %)	12 (24 %)	28 (18.7)	
	Non-smoker	44 (88 %)	40 (80 %)	39 (76 %)	122 (81.3 %)	NS
**Number of co-morbidities**	None	34 (68.0 %)	32 (64.0 %)	26 (52 %)	92 (61.3 %)	NS
	1	11 (22 %)	14 (28 %)	20 (40 %)	45 (30 %)	
	2	5 (10 %)	3 (6 %)	4 (8 %)	12 (8 %)	
	3 or more	0 (0 %)	1 (2 %)	0 (0 %)	1 (0.7 %)	
**Family History**	Yes	32 (64 %)	39 (78 %)	37 (74 %)	108 (72 %)	NS
	No	18 (36 %)	11 (22.0 %)	13 (26 %)	42 (28 %)	
**PHQ-9 overall score**	Moderate to severe	6 (12.2 %)	6 (12 %)	10 (20 %)	22 (14.8 %)	0.04
	Mild	19 (38.8 %)	10 (20 %)	21 (42 %)	50 (33.6 %)	
	Minimal	24 (49.2 %)	34 (68 %)	19 (38 %)	77 (51.7 %)	
**GAD-7 overall score**	Severe	4 (8 %)	1 (2 %)	2 (4 %)	7 (4.7 %)	NS
	Moderate	2 (4 %)	2 (4 %)	5 (10 %)	9 (6 %)	
	Mild	11 (22 %)	10 (20 %)	17 (34 %)	38 (25.3 %)	
	None to Minimal	33 (66 %)	37 (74 %)	26 (52 %)	96 (64 %)	

Mean ± SD for continuous variables or frequencies (column %) for categorical variables. Abbreviations: BMI: Body mass index; PHQ-9: Patient Health Questionnaire – 9; GAD-7: Generalized Anxiety Disorder-7; SD: Standard deviation.

Overall we found statistically significant differences between exposure groups in the distribution of the following variables: sex (p-value = 0.04), age (p-value <0.001), nationality (p-value = 0.001), level of education (p-value <0.001), marital status (p-value <0.004), BMI (p-value = 0.02), duration of diabetes (p-value <0.001), family support (p-value = 0.005), and overall PHQ-9 score (p-value = 0.04). We did not find statistically significant differences across type of diabetes for the following variables: employment status, smoking status, number of comorbidities, family history of diabetes mellitus, and GAD-7 overall score (Table 1).

### 3.2 Mean differences in PHQ-9 and GAD-7 scores by type of diabetes

There was a statistically significant difference in the overall mean PHQ-9 scores (p-value = 0.014). Post-hoc analysis revealed that a statistically significant difference existed between the pre-diabetes group and the T2DM group (Mean Difference = 2.46, 95 % CI: 0.4517 to 4.4683, p-value = 0.012). There was no statistically significant difference between the GAD-7 groups overall or in post-hoc analysis (p-value >0.05) (Table 2).

**Table 2:** Mean PHQ-9 and GAD-7 scores and type of diabetes mellitus.

PHQ-9				
Overall	Type 1 diabetes	Type 2 diabetes	Pre-diabetes	P-value
**PHQ-9**	5.61 ± 4.59	3.96 ± 3.57	6.42 ± 4.50	0.014
Between study group comparisons				
**Group comparison**	Mean difference in PHQ-9 score			**P-value**
**Pre-diabetes - Type 2 diabetes**	2.46, 95 % CI (0.452, 4.47)			0.012
**Type 1 diabetes – Type 2 diabetes**	1.65, 95 % CI (−0.37, 3.67)			NS
**Pre-diabetes – Type 1 diabetes**	0.80, 95 % CI (−1.21, 2.82)			NS
**GAD-7**				

**Overall**	Type 1 diabetes	Type 2 diabetes	Pre-diabetes	**P-value**

**GAD-7**	4.71 ± 3.74	5.63 ± 4.52	5.40 ± 3.94	NS
Between study group comparisons				
**Group comparison**	Mean difference in GAD-7 scores			**P-value**
**Pre-diabetes - Type 2 diabetes**	1.89, 95 % CI (−0.57, 4.36)			NS
**Type 1 diabetes – Type 2 diabetes**	2.06, 95 % CI (-0.24, 4.36)			NS
**Pre-diabetes – Type 1 diabetes**	−0.17, 95 % CI (−2.79, 2.45)			NS

NS = Not significant; p-value >0.05.

Between study group comparisons was conducted using Tukey's post-hoc test.

PHQ-9 = Patient Health Questionnaire-9, GAD-7: Generalized Anxiety Disorder-7.

### 3.3 Mean differences in PHQ-9 and GAD-7 scores by sex

Consistent findings occurred when examining within exposure group mean PHQ-9 and GAD-7 scores. There were statistically significant increased mean scores in females (PHQ-9: 6.72 ± 4.64, p-value = 0.003; GAD-7: 5.63 ± 4.52, p-value = 0.037) compared to males (PHQ-9: 3.17 ± 2.81; GAD-7: 2.28 ± 2.58) among individuals with type 2 diabetes mellitus. No statistically significant differences in the mean score for the PHQ-9 or GAD-7 were found when comparing males and females with T1DM or with pre-diabetes (Table 3).

**Table 3:** The mean difference assessed within-group sex differences in PHQ-9 and GAD-7 scores by study groups.

PHQ-9			
	Males	Females	p-value
**Type 1 diabetes**	5.41 ± (5.08)	5.90 ± (3.86)	NS
**Type 2 diabetes**	3.17 ± (2.81)	6.72 ± (4.64)	0.003
**Pre-diabetes**	6.91 ± (5.00)	6.00 ± (4.05)	NS
**GAD-7**			

	**Males**	**Females**	**p-value**

**Type 1 diabetes**	4.34 ± (4.97)	4.71 ± (3.74)	NS
**Type 2 diabetes**	2.28 ± (2.58)	5.63 ± (4.52)	0.037
**Pre-diabetes**	4.17 ± (4.36)	5.40 ± (3.94)	NS

NS = Not significant; p-value >0.05.

Within study group comparisons was conducted using independent samples *t*-test.

PHQ-9 = Patient Health Questionnaire-9, GAD-7: Generalized Anxiety Disorder-7.

## 4 Discussion

Results from this study found the prevalence of moderate to severe depressive symptoms to be 12 % in T2DM patients, 12.2 % in T1DM patients, and 20 % in individuals with pre-diabetes. A similar pattern emerged with the prevalence of moderate to severe anxiety symptoms with T2DM patients (6 %) having the lowest prevalence followed by T1DM patients (12 %), and with the pre-diabetes group having the highest prevalence (14 %). Moreover, we found people living with diabetes are a heterogeneous group with different characteristics showing significant differences based on the type of diabetes and pre-diabetes for sex, age, nationality, level of education, marital status, BMI, duration of diabetes, and family support. Our results further indicated that there is a significant difference in the overall PHQ-9 score by type of diabetes which is consistent with a recently published paper in Qatar that examined depressive symptoms in Qatari patients living with diabetes only [27]. Interestingly, we found poorer PHQ-9 scores in type 1 diabetes patients and individuals with pre-diabetes. A systematic review indicated that patients with type 1 diabetes are more than 3 times more likely to develop depressive symptoms compared to type 2 diabetes which is similar to our findings [28]. While we did not find a statistically significant difference in the GAD-7 score, the presence of GAD symptoms (GAD-7 score ≥10) was recently reported to be independently associated with a two-fold increased risk of type 2 diabetes [29] suggesting its value in screening patients with pre-diabetes. Finally, to the best of our knowledge, examining PHQ-9 scores among individuals with pre-diabetes, has not been well reported in the scientific literature.

### 4.1 Pre-diabetes and depressive/anxiety symptoms

When examining pre-diabetes and depressive or anxiety symptoms, the pre-diabetes group had the highest PHQ-9 scores compared to type 1 and type 2 diabetes indicating increased depressive symptoms (20 %). Previous studies found pre-diabetes being associated with an increased risk of depression [30] and a higher prevalence of depressive symptoms [31]. This is an important finding because lifestyle modification is a keystone element of pre-diabetes management to reduce the transition to type 2 diabetes [32] as well as part of type 2 diabetes management. A prospective cohort study that assessed whether depressive or anxiety symptoms in individuals with pre-diabetes increases the risk of developing type 2 diabetes found that individuals with high depression and pre-diabetes were 2.8 times more likely to develop diabetes compared to pre-diabetes alone, while those with anxiety symptoms and prediabetes were 2.4 times more likely to develop diabetes compared to those with pre-diabetes alone [32]. These findings indicate a synergistic relationship between depressive symptoms and pre-diabetes in the development of diabetes [33] and highlight the need for screening pre-diabetes patients for both depression and anxiety.

### 4.2 Type 2 diabetes and depressive/anxiety symptoms

A recent study found depression negatively impacts compliance to lifestyle modifications in type 2 diabetes patients [34]. While a previous meta-analysis found similar results, but moderator analysis specifically indicated that self-care behaviors and missed medical appointments were negatively impacted by depression [35]. Altogether, lack of adherence to diabetes management leads to an increased risk of additional complications associated with type 2 diabetes leading to poorer health outcomes and decreased quality of life [36]. Our results found patients with type 2 diabetes had better PHQ-9 outcomes compared to pre-diabetes patients, however, there still remained 12 % classified with moderate to severe depressive symptoms. This proportion is significantly lower than the previously reported prevalence of 25 % in the United States, which requires further study [37]. The literature indicates a bi-directional relationship between depression and type 2 diabetes [38]. Moreover, co-morbid major depressive disorder (the most prevalent mental health disorder in the US) and type 2 diabetes result in increased mortality, and development of type 2 diabetes, increased recurrence of depression, and poorer treatment compliance for either condition [37]. It should be noted our study is cross-sectional, therefore we are unable to assess the directionality of the relationship between depressive symptoms and diabetes. Irrespective we found consistent results with the previous literature and examining the directionality and impact of treatment warrants further investigation.

### 4.3 Sex differences in diabetes and depressive/anxiety symptoms

In our analysis, we found females with type 2 diabetes reported statistically significant higher mean scores for both anxiety and depression symptoms compared to males but not in other groups. In Qatar, studies have shown women with diabetes were 1.8 times more likely to have depression compared to men [27,39,40]. Furthermore, a cohort study with a 20 year follow-up that examined sex differences in the relationship between depression and diabetes found that women with increased depressive symptoms, but not men were more likely to develop diabetes, while with anxiety there was a similar relationship but not as marked [41]. There are several potential mechanisms linking sex differences with mental health disorders and incident diabetes including different coping mechanisms, inflammatory responses, or hypothalamus-pituitary axis dysregulation [41].

Important to note that we did not find statistically significant differences in the PHQ-9 or GAD-7 scores within female groups (when comparing females with type 1 diabetes to type 2 diabetes to pre-diabetes groups). However, among males, we found higher depressive scores among the pre-diabetes group compared to type 2 diabetes. This may be due to increased statistical power attributed to our sample, where we recruited 39 males, and 11 females with type 2 diabetes. However, the sex-based disparities could also be related to stigmatization associated with mental health in Qatar. A previous study aimed to assess the stigma associated with mental health disorders reported that men had higher stigmatization scores compared to women for mental health disorders [42]. As a result, females might be more willing to speak and express feelings to a friend or family member much more readily than males who tend to avoid discussing emotions and feelings partially attributed to gender roles [28,43].

### 4.4 Limitations

There were several limitations to the study. Respondent bias may have influenced our results but to best address this issue, all data collection was conducted by the researcher in either English or Arabic; whichever the participant preferred. The site chosen (QDA) is a non-profit organization that aims to provide the best diabetes education and consultation services for patients living with diabetes and those who are at risk to develop diabetes. They have a large client base from different areas of Qatar, which facilitated more representative recruitment. However, while validated previously, there is no study examining whether scores in English and Arabic can be combined. Although, a previous study found that English and French scores for the PHQ-9 can be combined [44]. Second, there exists a stigma associated with mental health disorders leading to potential under-reporting. To best approach this sensitive topic, the researcher was trained to provide neutral responses and to be non-judgmental to mitigate feelings of stigmatization when participants answered questions. In addition, results of our sample groups indicated statistically significant differences in the demographics of the sample by exposure group. Finally, this study was cross-sectional therefore causation or directionality of the relationship between diabetes and depression or anxiety cannot be inferred.

## 5 Conclusion

The results of our study found patients living with pre-diabetes, and females with T2DM are potentially vulnerable populations who should be screened for mental health disorders. We recommend early screening for mental health disorders not just for people living with diabetes, but especially for those who are diagnosed with pre-diabetes and at risk of developing the disease.

## Funding

This research did not receive any specific grant from funding agencies in the public, commercial, or not-for-profit sectors.

## CRediT authorship contribution statement

**Montaha Mahmoud:** Conceptualization, Data curation, Formal analysis, Investigation, Methodology, Writing – original draft, Writing – review & editing. **Razi Mahmood:** Conceptualization, Data curation, Formal analysis, Funding acquisition, Investigation, Methodology, Project administration, Resources, Software, Supervision, Writing – original draft, Writing – review & editing.

## Declaration of competing interest

The authors declare that they have no known competing financial interests or personal relationships that could have appeared to influence the work reported in this paper.
